# Inhibition of FGF receptor blocks adaptive resistance to RET inhibition in *CCDC6-RET*–rearranged thyroid cancer

**DOI:** 10.1084/jem.20210390

**Published:** 2022-05-05

**Authors:** Renuka Raman, Jacques A. Villefranc, Timothy M. Ullmann, Jessica Thiesmeyer, Viviana Anelli, Jun Yao, James R. Hurley, Chantal Pauli, Rohan Bareja, Kenneth Wha Eng, Princesca Dorsaint, David C. Wilkes, Shaham Beg, Sarah Kudman, Reid Shaw, Michael Churchill, Adnan Ahmed, Laurel Keefer, Ian Misner, Donna Nichol, Naveen Gumpeni, Theresa Scognamiglio, Mark A. Rubin, Carla Grandori, James Patrick Solomon, Wei Song, Juan Miguel Mosquera, Noah Dephoure, Andrea Sboner, Olivier Elemento, Yariv Houvras

**Affiliations:** 1 Department of Surgery, Weill Cornell Medical College, New York, NY; 2 Department of Medicine, Weill Cornell Medical College, New York, NY; 3 Department of Pathology and Molecular Pathology, University Hospital Zurich, Zurich, Switzerland; 4 The Caryl and Israel Englander Institute for Precision Medicine and the Institute for Computational Biomedicine, Weill Cornell Medical College, New York, NY; 5 Department of Pathology and Laboratory Medicine, Weill Cornell Medical College, New York, NY; 6 SEngine Precision Medicine, Seattle, WA; 7 Department of Biochemistry, Weill Cornell Medical College, New York, NY; 8 Personal Genome Diagnostics, Inc., Baltimore, MD; 9 Department of Radiology, Weill Cornell Medical College, New York, NY; 10 Bern Center for Precision Medicine, University of Bern, Bern, Switzerland; 11 Sandra and Edward Meyer Cancer Center, Weill Cornell Medical College, New York, NY

## Abstract

Genetic alterations in *RET* lead to activation of ERK and AKT signaling and are associated with hereditary and sporadic thyroid cancer and lung cancer. Highly selective RET inhibitors have recently entered clinical use after demonstrating efficacy in treating patients with diverse tumor types harboring *RET* gene rearrangements or activating mutations. In order to understand resistance mechanisms arising after treatment with RET inhibitors, we performed a comprehensive molecular and genomic analysis of a patient with *RET*-rearranged thyroid cancer. Using a combination of drug screening and proteomic and biochemical profiling, we identified an adaptive resistance to RET inhibitors that reactivates ERK signaling within hours of drug exposure. We found that activation of FGFR signaling is a mechanism of adaptive resistance to RET inhibitors that activates ERK signaling. Combined inhibition of FGFR and RET prevented the development of adaptive resistance to RET inhibitors, reduced cell viability, and decreased tumor growth in cellular and animal models of *CCDC6-RET*–rearranged thyroid cancer.

## Introduction

Resistance to small-molecule kinase inhibitors is a critical problem in cancer treatment. Cancer cells engage multiple strategies to evade treatment (Vasan et al., 2019). Prolonged exposure to kinase inhibitors, including kinases that activate ERK signaling, leads to acquired genetic alterations that confer resistance. Increasingly it has been recognized that cancer cells undergo a rapid adaptation, within hours of exposure to ERK pathway kinase inhibitors, as a consequence of disrupting negative feedback mechanisms (Chandarlapaty, 2012). Adaptive resistance endows cancer cells with the ability to reactivate ERK signaling and may be responsible for the incomplete initial response of solid tumors to treatment with kinase inhibitors.

Understanding the specific mechanisms subverted in cancer cells by kinase inhibitors remains critical for improving clinical outcomes. Adaptive resistance to kinase inhibitors has been documented in diverse cellular and genetic contexts. In *BRAF*-mutant melanoma, treatment with BRAF inhibitors initially inhibits ERK signaling, followed by a rebound activation of ERK signaling as negative feedback pathways are disrupted (Lito et al., 2012). In *BRAF*-mutant colorectal cancer, BRAF inhibitors trigger resistance through an alternative mechanism, as EGFR signaling is upregulated (Prahallad et al., 2012; Corcoran et al., 2012). Inhibition of MEK has been associated with activation of alternative signaling pathways, including PI3K/AKT (Turke et al., 2012), and upregulation of alternative receptor tyrosine kinases (Sun et al., 2014). Recent studies have implicated SHP2, a dual-specificity phosphatase, as a mediator of resistance to MEK and KRAS inhibitors and suggested broad application of SHP2 inhibitors in defeating adaptive resistance mechanisms (Dardaei et al., 2018; Fedele et al., 2018; Ahmed et al., 2019). A detailed understanding of adaptive resistance mechanisms has led to combination therapies to defeat tissue-specific resistance mechanisms and has led to improved clinical outcomes (Long et al., 2014; Kopetz et al., 2019).

Chromosomal rearrangements involving receptor tyrosine kinases are an increasingly recognized class of driver mutations in solid tumors. Rearrangements involving *ALK*, *ROS1*, *NTRK* family genes, and *RET* are found in lung, colon, thyroid, and other cancers (Farago and Azzoli, 2017). Several small-molecule kinase inhibitors targeting fusion oncoproteins have demonstrated efficacy in treating solid tumors harboring gene rearrangements (Blackhall et al., 2017; Drilon et al., 2017). Nonselective RET inhibitors were first shown to be active in treatment of medullary thyroid cancer (MTC), where they are disease controlling but not curative (Elisei et al., 2013; Wells et al., 2010). Recently two new selective RET inhibitors, selpercatinib and pralsetinib, have shown significant efficacy in treating patients with RET gene rearrangements and activating mutations, in both lung and thyroid cancers (Wirth et al., 2020; Drilon et al., 2020). While treatment with new selective RET inhibitors has been associated with significant clinical responses, a majority of patients experience a partial response or disease stabilization as their best clinical outcome. Understanding the mechanisms of drug resistance in the treatment of RET-dependent cancers is critically important.

*RET* is subject to multiple genetic alterations that activate it as an oncogene in diverse cell types (reviewed in Santoro and Carlomagno, 2013). In the hereditary syndrome of multiple endocrine neoplasia type 2, germline mutations in *RET* predispose patients to MTC and pheochromocytoma (Donis-Keller et al., 1993; Santoro et al., 1995). Patients with sporadic MTC predominantly harbor point mutations in *RET*, generally in the kinase domain. Chromosomal rearrangements that result in *RET* gene fusions lead to aberrant expression of a RET kinase domain that signals through ERK and PI3K/AKT pathways. *RET* fusions have been identified in 5–10% of papillary thyroid cancers and 1–2% of lung cancers and involve distinct fusion partners, including *KIF5B* in lung adenocarcinoma and *CCDC6* in thyroid and lung cancer (Grieco et al., 1990; Kohno et al., 2012; Takeuchi et al., 2012; Lipson et al., 2012). *RET* fusions are mutually exclusive with *BRAF* and *RAS* gene mutations in thyroid cancer, consistent with its role as a driver oncogene. Mutations in the *TERT* promoter have been identified in thyroid cancer, including in *RET*-rearranged thyroid cancer, but their association with disease progression has been unclear. Both nonselective and selective RET inhibitors have been associated with the development of acquired genetic mutations leading to drug resistance (Dagogo-Jack et al., 2018; Solomon et al., 2020; Zhu et al., 2020). Based on our experience treating *RET*-rearranged papillary thyroid cancer, we suspected that an incomplete clinical response to RET inhibition may be due to adaptive resistance leading to rebound ERK activation. We sought to identify the mechanism of adaptive resistance to RET inhibitors and to nominate a drug combination capable of overcoming resistance.

## Results

**Figure 1.**
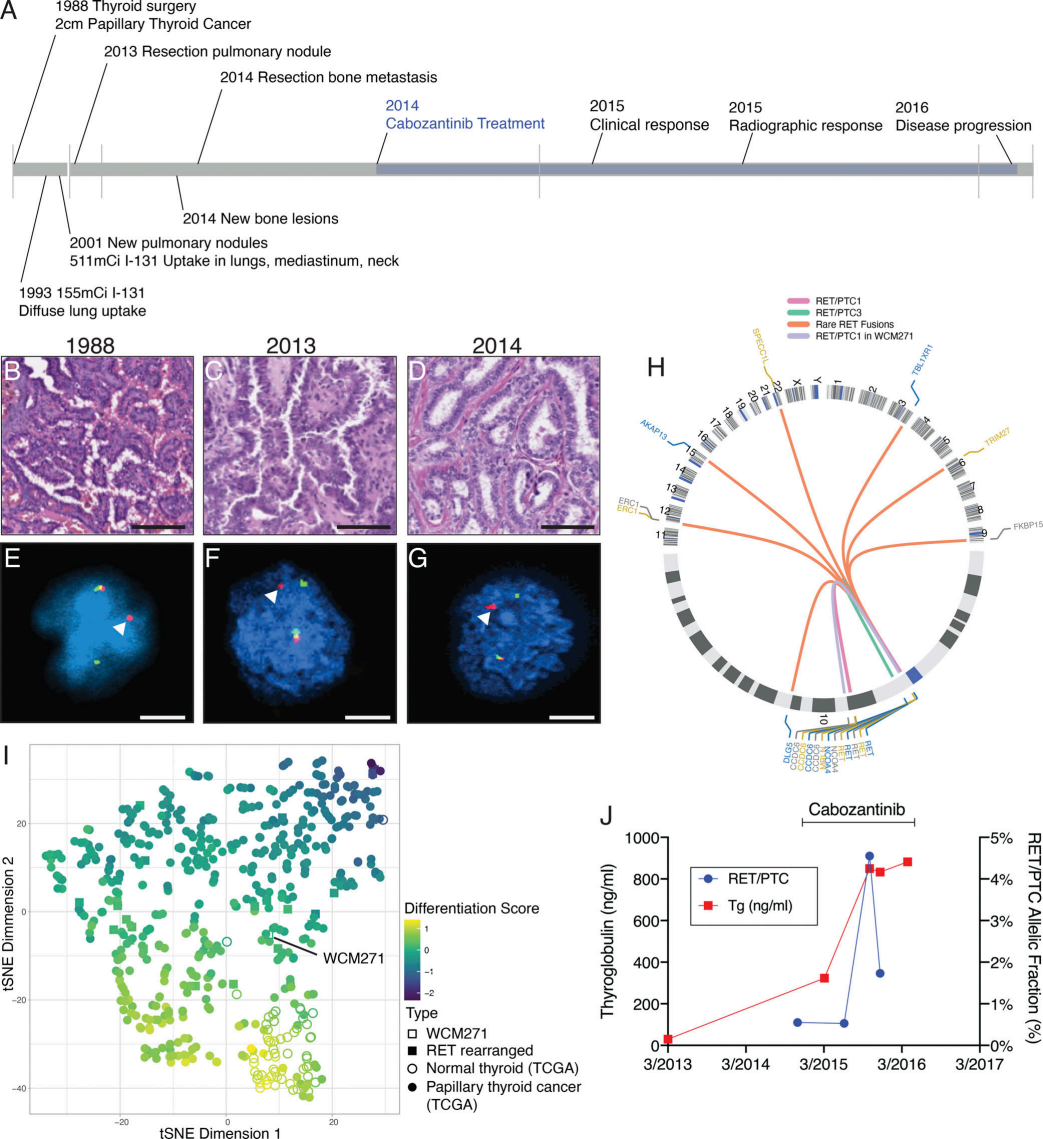
**Molecular and genomic characterization identifies a RET rearrangement in a patient with a three-decade history of papillary thyroid cancer. (A)** Timeline from diagnosis (1988) to relapse and treatment with a RET inhibitor. Treatment with radioactive iodine; imaging results are summarized. Clinical timeline between 1988 and 2013 is compressed for clarity. **(B–G)** Histopathology (B–D; scale bar = 100 μm) and FISH (E–G; scale bar = 20 μm) from biopsy specimens obtained at the time of diagnosis in 1988 (B and E), from a pulmonary metastasis in 2013 (C and F), and from a bone metastasis in 2014 (D and G). Chromosomal rearrangement involving the *RET* gene was confirmed by FISH; arrowheads indicate rearrangement (E–G). In E**–**G, representative images from three replicates are shown. **(H**) Circos plot diagramming the index patient’s intrachromosomal rearrangement involving *RET* and *CCDC6* (blue, chr10) overlayed with *RET* rearrangements identified in the TCGA papillary thyroid cancer cohort (*n* = 22). **(I)** tSNE (t-distributed stochastic neighbor embedding) analysis of gene expression comparing papillary thyroid cancers (TCGA) to the index patient (WCM271). Thyroid differentiation score is indicated by color; *RET* rearrangement status is indicated by icon. **(J)** Serum thyroglobulin (Tg, red) and *CCDC6-RET* allele fraction (blue) determined from circulating tumor cells are plotted over the indicated time; treatment with cabozantinib is indicated.

We performed a comprehensive molecular, genetic, and genomic analysis of an individual patient (WCM271) with a three-decade history of thyroid cancer who enrolled in a research protocol for precision medicine at our institution. A review of the patient’s cancer treatment history revealed the development of radioiodine refractory thyroid cancer metastatic to lung (13 yr) and bone (26 yr) after surgery to remove the primary tumor (Fig. 1 A). A molecular analysis of tumor tissue from metastatic lesions revealed a rearrangement involving the *RET* gene, and the patient was subsequently treated with cabozantinib (XL184), a nonselective RET inhibitor. This patient provided a unique opportunity to examine the efficacy of RET inhibition.

**Figure S1.**
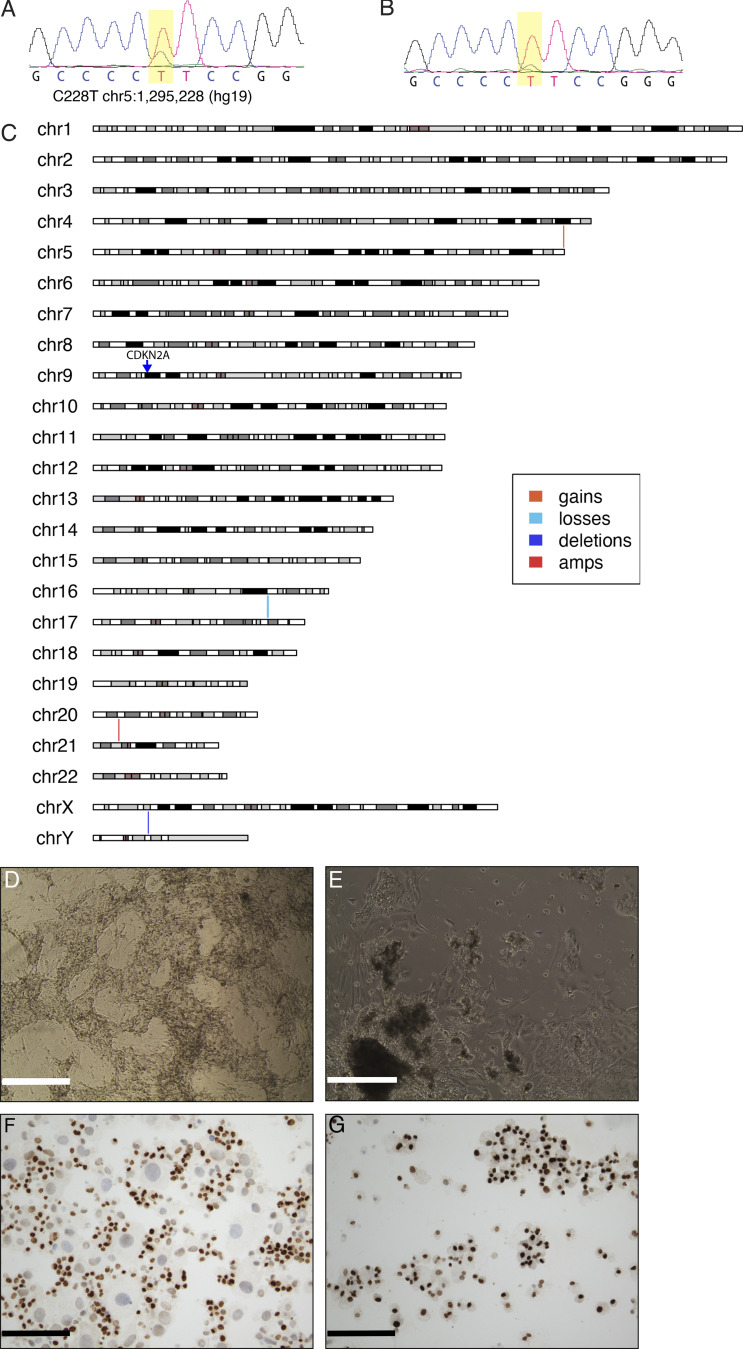
**Genetic alterations identified in WCM271 metastatic lesions; characterization of primary tumor cells. (A and B)** Sanger sequencing of *TERT* promoter performed on DNA from bone metastasis (A) or lung metastasis (B). **(C)** Copy number alterations in WCM271 are indicated on an ideogram of human chromosomes. Vertical lines represent copy number alterations as indicated in the legend. **(D)** Primary tumor cells isolated from bone metastasis cultured on mitomycin-treated mouse feeder cells; scale bar = 400 μm. **(E)** Primary tumor cells cultured on gelatin-coated plates; scale bar = 400 μm. **(F)** Immunohistochemistry for TTF1 on tumor cells isolated from D; scale bar = 100 μm. **(G)** Immunohistochemistry for TTF1 on tumor cells isolated from E; scale bar = 100 μm. Immunohistochemistry was performed on 50,000 cells using anti-TTF1 mAB (Neomarkers Thermoscientific, 1:200). TTF1^+^ immunostaining may identify cells of thyroid or lung origin.

**Figure S2.**
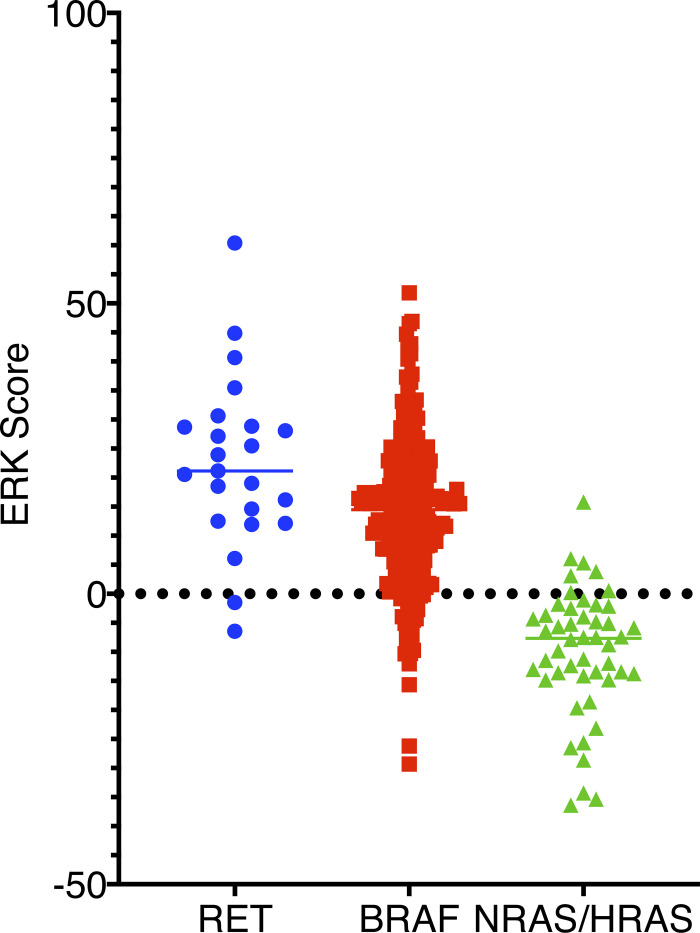
**ERK score in papillary thyroid cancers from TCGA cohort.** We compared ERK scores between RET-rearranged (*n* = 23), BRAF mutant (*n* = 240), and HRAS/NRAS mutant (*n* = 48) papillary thyroid cancers. RET-rearranged samples have a significantly higher mean ERK score than either BRAF (P = 0.01) or RAS mutant (P = 5.7 × 10^−11^) cancers; Student’s *t* test or ANOVA.

A review of the patient’s treatment history revealed evidence of microscopic radioiodine avid disease 5 yr after resection of a primary papillary thyroid cancer. We confirmed the presence of a *RET* gene rearrangement from the patient’s primary tumor (1988) using fluorescence in situ hybridization (FISH; Fig. 1 E). We performed targeted hybrid capture-based next-generation sequencing on DNA extracted from the patient’s primary tumor and a lymph node metastasis. No additional mutations were identified. Analysis of metastatic tumor tissue isolated from lung (Fig. 1 C) and bone (Fig. 1 D) specimens confirmed the presence thyroid cancer and a *RET* gene rearrangement (Fig. 1, F and G). Histologic assessment of the bone and lung metastases revealed papillary thyroid carcinoma with foci of necrosis and increased mitotic activity, consistent with high-grade features that were not present in the primary tumor. A molecular analysis performed on bone metastasis identified the presence of a *TERT* promoter variant and a loss of *CDKN2A* (Fig. S1, A–C). No additional driver mutations were identified from bone or lung metastases using whole-exome sequencing. We expanded primary tumor cells isolated from a resected metastasis in short-term culture, and we confirmed a thyroid cell of origin (Fig. S1, D–G). We performed next-generation RNA sequencing on WCM271 tumor tissue and identified CCDC6 as the RET gene fusion partner. RET fusions with CCDC6 lead to a constitutively active cytoplasmic protein. The Cancer Genome Atlas (TCGA) papillary thyroid cancer cohort contains a well-annotated set of 402 primary papillary thyroid cancers, including 25 RET-rearranged cases (Cancer Genome Atlas Research, 2014). We compared WCM271 to *RET*-rearranged thyroid cancers in the TCGA cohort (Fig. 1 H) and the entire cohort (Fig. 1 I). We found that *RET*-rearranged samples in the TCGA dataset exhibited significant heterogeneity in gene expression and thyroid differentiation. The index patient’s tumor had an intermediate thyroid differentiation score and clustered with other *RET*-rearranged papillary thyroid cancers (Fig. 1 I). We compared the ERK score, a measure of ERK activity derived from RNAseq data, from *RET*-rearranged, BRAF mutant, and RAS mutant papillary thyroid cancers in TCGA patients, and we found that *RET*-rearranged cancers have significantly higher ERK scores than either BRAF or NRAS/HRAS mutant tumors (Fig. S2).

Based on the finding of a RET rearrangement, we treated the patient with cabozantinib (XL184), a nonselective RET inhibitor known to have antitumor activity in differentiated thyroid cancer (Cabanillas et al., 2014). Treatment was associated with clinical improvement in bone pain and a radiographic response (35% decrease in tumor volume of a sternal lesion after 9 mo of treatment). Serum thyroglobulin, a tumor marker in thyroid cancer, was elevated and stabilized during the treatment period (Fig. 1 J). Using cell-free DNA (cfDNA) isolated from serum during the treatment period, we observed a decrease in *RET* fusion gene allele fraction during treatment (Fig. 1 J). Treatment response to cabozantinib in the index patient lasted 9 mo and was followed by disease progression. Because the patient experienced a partial response to treatment of limited duration, we sought to determine the mechanism of resistance to RET inhibition. Despite substantial experimental effort, we were unable to establish a primary cell line, organoid model, or xenograft in immunocompromised mice; therefore we used established cancer cell lines harboring *RET* rearrangements to model drug resistance.

**Figure 2.**
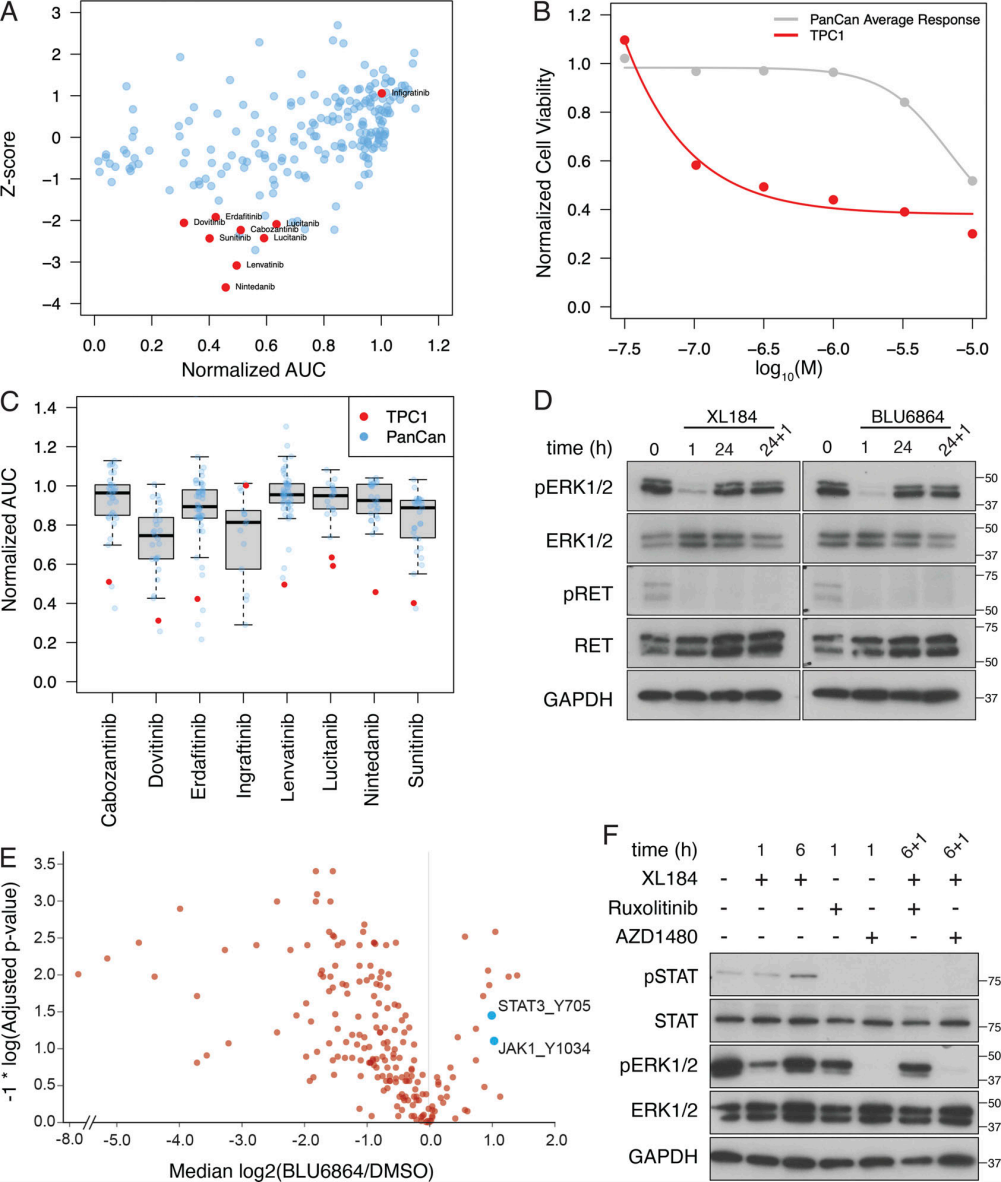
**Drug screening and phosphotyrosine proteomic profiling to characterize response to RET inhibition in TPC1 cells. (A)** Results from a drug screen testing 209 compounds in TPC1 cells for effects on viability. Select kinase inhibitors are highlighted in red, including cabozantinib. AUC, area under the curve. **(B)** Short-term cellular viability after exposure to cabozantinib in TPC1 cells (red) is compared to the average dose response of a panel of cancer cell lines (PanCan, gray, *n* = 52). **(C)** Results from a drug screen comparing TPC1 cells (red) to a panel of 52 cancer cell lines (gray) after treatment with cabozantinib and select kinase inhibitors. **(A–C)** The drug screens performed were replicated in two independent experiments, and a representative experiment is shown. **(D)** Western blot analysis of ERK activation and RET phosphorylation in TPC1 cells treated with 100 nM cabozantinib (XL184) or 100 nM BLU6864 (RETi). Cells were harvested at the indicated times; the 24 + 1 timepoint indicates a treatment of an additional 1 h with the indicated compound after an initial 24 h of treatment. Representative blots are shown from four independent experiments. **(E)** TPC1 cells treated with BLU6864 were compared to vehicle-treated cells, and phosphotyrosine peptides were analyzed by mass spectrometry. Peptides corresponding to JAK1 (Y1034) and STAT3 (Y705) are highlighted. **(F)** Western blot analysis of JAK and ERK activation in TPC1 cells treated with XL184, ruxolitinib, or AZD1480 (JAK2i). Treatment time is indicated, the 6 + 1 timepoint indicates a treatment of 1 h with either ruxolitinib (JAK2i; lane 6) or AZD1480 (lane 7) after a 6-h treatment with XL184. Representative blots are shown from three independent experiments.

Using TPC1 cells, a human thyroid cancer cell line expressing a CCDC6-RET fusion protein (Ishizaka et al., 1989), we performed a series of drug screens to identify insights into drug sensitivity and resistance. We found that TPC1 cells are sensitive to multiple kinase inhibitors, including cabozantinib (Fig. 2 A). We found that TPC1 cells are significantly more sensitive to cabozantinib than a panel of human cancer cell lines (Fig. 2 B). We examined the relative sensitivity of TPC1 cells to top scoring kinase inhibitors, and we found that TPC1 cells are significantly more sensitive than the majority of cell lines tested (Fig. 2 C). We observed that several kinase inhibitors that scored positive in these assays had significant anti-FGFR activity, including nintedanib, erdafitinib, and dovitinib.

**Figure S3.**
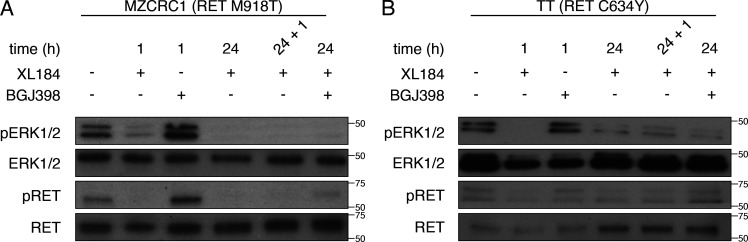
**Biochemical signaling in MZCRC1 and TT cells after exposure to cabozantinib. (A)** MZCRC1 cells were exposed to the indicated compounds, and Western blots were performed on cell lysates. **(B)** TT cells were exposed to the indicated compounds, and Western blots were performed on cell lysates. Representative image from three independent experiments. For the 24 + 1 treatment condition, fresh drug was added after 24 h of exposure, and a lysate was prepared after 1 h.

We next studied the biochemical effects of treatment with RET inhibitors in TPC1 cells. We found that exposure to either cabozantinib (XL184), a nonselective RET inhibitor, or BLU6864, a selective RET inhibitor, is associated with rapid inhibition of ERK signaling (Fig. 2 D). After longer exposure, TPC1 cells exhibited a rebound in phospho-ERK (24 h) and were then refractory to repeat treatment with RET inhibitors (24 + 1 h). Both RET inhibitors decreased RET phosphorylation, which did not rebound. We confirmed a rebound in pERK after RETi using a second RET fusion thyroid cancer cell line, CUTC48 (Fig. S5 A). To determine if RET inhibition triggers adaptive resistance in other RET mutant cellular contexts we tested two MTC cell lines, a neuroendocrine cancer associated with activating mutations in RET (Cooley et al., 1995). Mzcrc1 cells harbor an activating mutation in the RET kinase domain (M918T). Treatment of MZCRC1 cells with XL184 inhibited ERK activation and was not associated with a rebound in ERK activation (Fig. S3 A). TT cells harbor an extracellular mutation (C634Y) that leads to ligand independent receptor dimerization. Treatment of TT cells with XL184 inhibited ERK activation that rebounded slightly at 24 h (Fig. S3 B). These results indicate that RET-rearranged cells undergo an adaptive resistance to RET inhibition that is unique compared with MTC cell lines harboring point mutations in RET.

In an effort to identify signaling pathways responsible for mediating adaptive resistance we performed proteome profiling of phosphotyrosine using mass spectrometry. TPC1 cells treated with BLU6864 were profiled 24 h after treatment to identify changes in the abundance of phosphotyrosine containing peptides. While the majority of phosphotyrosine peptides showed a decrease in abundance compared with vehicle treatment, we identified several peptides with an increase in phosphotyrosine abundance, including JAK1 and STAT3 (Fig. 2 E). We tested ruxolitinib, a highly specific JAK inhibitor, and we found that exposure to ruxolitinib is associated with inhibition of JAK activity as measured by pSTAT but had no significant effect on ERK signaling (Fig. 2 F). Combined treatment with cabozantinib and ruxolitinib was ineffective in preventing a rebound in ERK activation. Since JAK/STAT signaling is downstream of FGFR signaling (Dudka et al., 2010), and multiple FGFR inhibitors were identified in our drug screens, we tested AZD1480, a kinase inhibitor with both anti-JAK and anti-FGFR activity (Scuto et al., 2011). Treatment of TPC1 cells with AZD1480 abrogated both JAK activity and ERK signaling and was effective in blocking adaptive resistance to RET inhibition (Fig. 2 F).

**Figure 3.**
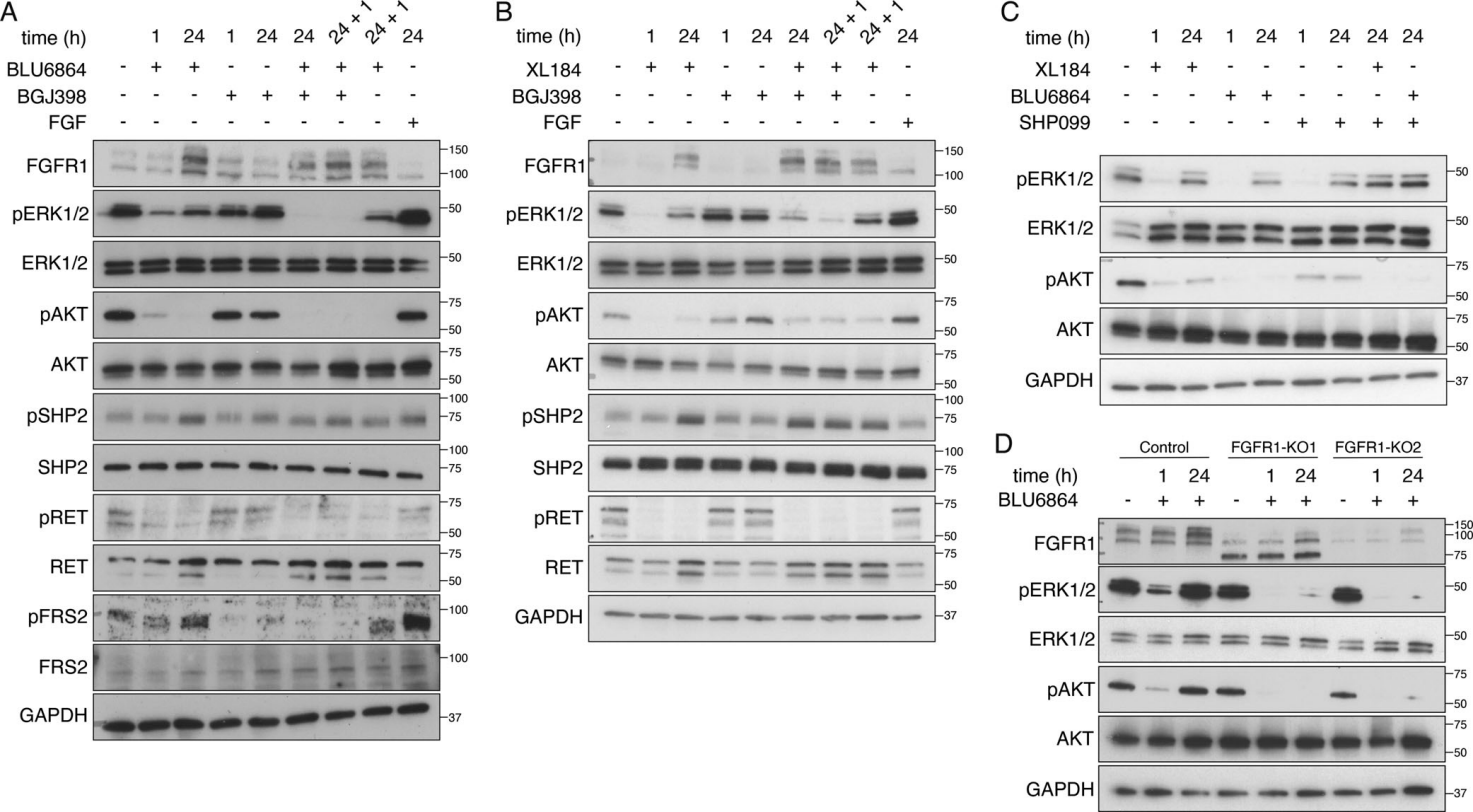
**Inhibition of FGFR abrogates adaptive resistance to RET inhibitors in TPC1 cells. (A)** Western blot analysis of FGFR1 and ERK activation and downstream signaling effectors in TPC1 cells treated with BLU6864 (RETi) alone or in combination with BGJ398 (FGFRi) over the indicated time course. **(A)** 24 + 1 timepoint indicates that the BGJ398 was added for 1 h after an initial incubation in BLU6864 for 24 h. **(B)** Western blot analysis of FGFR1 and ERK activation and downstream signaling effectors in TPC1 cells treated with XL184 alone or in combination with BGJ398 over the indicated time course. **(A)** 24 + 1 timepoint indicates that the BGJ398 was added for 1 h after an initial incubation in XL184 for 24 h. **(C)** Western blot analysis of ERK activation and downstream signaling effectors in TPC1 cells treated with XL184, BLU6864, and SHP099 alone or in combination. **(D)** Western blot analysis of FGFR1 and ERK activation in control infected TPC1 cells and in TPC1 cell clones isolated and expanded after lentiviral infection with sgRNAs targeting FGFR1 for genetic inactivation (FGFR1-KO1, FGFR1-KO2). Cells were treated with vehicle or BLU6864 for the indicated time and at a final concentration of 100 nM. For Western blots in A**–**D, a representative blot is shown from a minimum of three independent experiments.

To further examine the role of FGFR signaling in TPC1 cells, we used BGJ398, a small molecule with high selectivity and potency for FGFR 1–3 (Guagnano et al., 2011). TPC1 cells treated with BLU6864 showed an increase in FGFR1 at 24 h that paralleled the increase in pERK (Fig. 3, A, B, and D). TPC1 cells treated with BGJ398 alone showed a decrease in pFRS2, consistent with an on-target effect, but no significant inhibition of either ERK or AKT signaling pathways. Combined treatment with BLU6864 (RETi) and BGJ398 (FGFRi) effectively abrogated adaptive resistance and led to a decrease in ERK signaling (Fig. 3 A), despite persistently elevated levels of FGFR1. This pattern was also observed in TPC1 cells treated with XL184 (Fig. 3 B). Abrogation of adaptive resistance to RET inhibition occurred with either cotreatment with BGJ398 or addition of BGJ398 for 1 h after initial treatment with a RET inhibitor (24 + 1 h). We observed subtle differences between BLU6864 and XL184 in combination with BGJ398, including a more significant decrease in AKT signaling with BLU6864 with combined treatment. We examined the role of SHP2, a dual-specificity phosphatase implicated in RTK signaling and adaptive resistance to MEK inhibition. We found that SHP2 phosphorylation increases in TPC1 cells after treatment with RET inhibitors and remains elevated after treatment with BGJ398 (Fig. 3, A and B). Inhibition of SHP2 phosphatase activity using SHP099, an allosteric inhibitor of SHP2 (Garcia Fortanet et al., 2016), was associated with a decrease in ERK and AKT signaling but was not sufficient to abrogate adaptive resistance induced by RET inhibition in TPC1 cells (Fig. 3 C). To determine whether FGFR1 mediated adaptive resistance to BLU6864, we performed lentiviral infection and CRISPR/Cas9 gene editing targeting FGFR1 for genetic inactivation. We confirmed genetic inactivation of FGFR1 in individual lentiviral-infected clones (Fig. S5 B). Biochemical analysis of TPC1 clones harboring genetic inactivation of FGFR1 revealed that treatment with BLU6864 was not associated with rebound activation of pERK or pAKT at 24 h (Fig. 3 D). These data indicate that treatment with an FGFR inhibitor or genetic inactivation of FGFR1 blocks adaptive resistance caused by RET inhibition in TPC1 cells.

**Figure 4.**
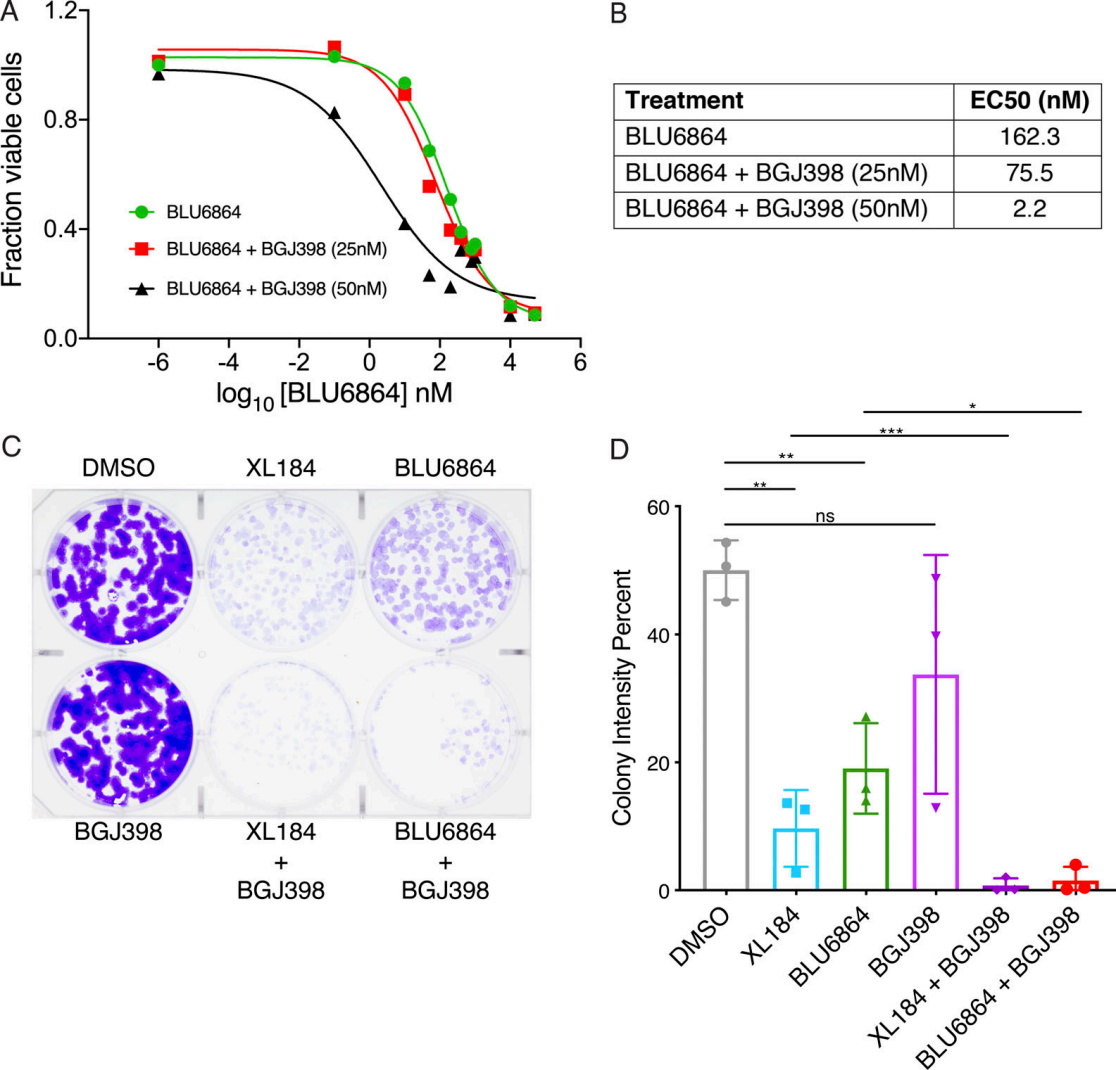
**FGFR inhibition sensitizes TPC1 cells to RET inhibitors. (A)** Short-term proliferation assay (48 h) of TPC1 cells treated with 100 nM BLU6864 alone or in combination with indicated doses of BGJ398; representative dose–response curves are shown from three independent experiments. **(B)** EC_50_ values of the indicated drug combinations as determined from A. **(C)** Long-term proliferation assay of TPC1 cells treated with XL184 or BLU6864 alone or in combination with BGJ398. Cells were treated for 7 d at 250 nM and stained with crystal violet. Three independent experiments were performed, and a representative plate is displayed. **(D)** Quantification of long-term viability of TPC1 cells treated with the indicated compounds was performed by calculating colony intensity; the mean and SD are displayed for technical replicates from one of three biological replicates. *, P < 0.05; **, P < 0.01; ***, P < 0.001, Student’s *t* test.

We examined the effect of combined RET and FGFR inhibition in TPC1 cells on growth and viability. Combined treatment with BGJ398 rendered TPC1 cells more sensitive to BLU6864 in short-term viability assays (Fig. 4 A) and decreased the half-maximal effective concentration (EC_50_) for BLU6864, consistent with synergy. In long-term viability assays, combined treatment with BGJ398 and either BLU6864 or XL184 was associated with decreased colony formation (Fig. 4, C and D). Similarly, in TPC1 cells with genetic inactivation of FGFR1, we found increased sensitivity to BLU6864 and decreased colony formation (Fig. S5, C and D). These data indicate that combined inhibition of FGFR and RET, or genetic inactivation of FGFR1, leads to decreased viability in RET-rearranged TPC1 cells.

**Figure 5.**
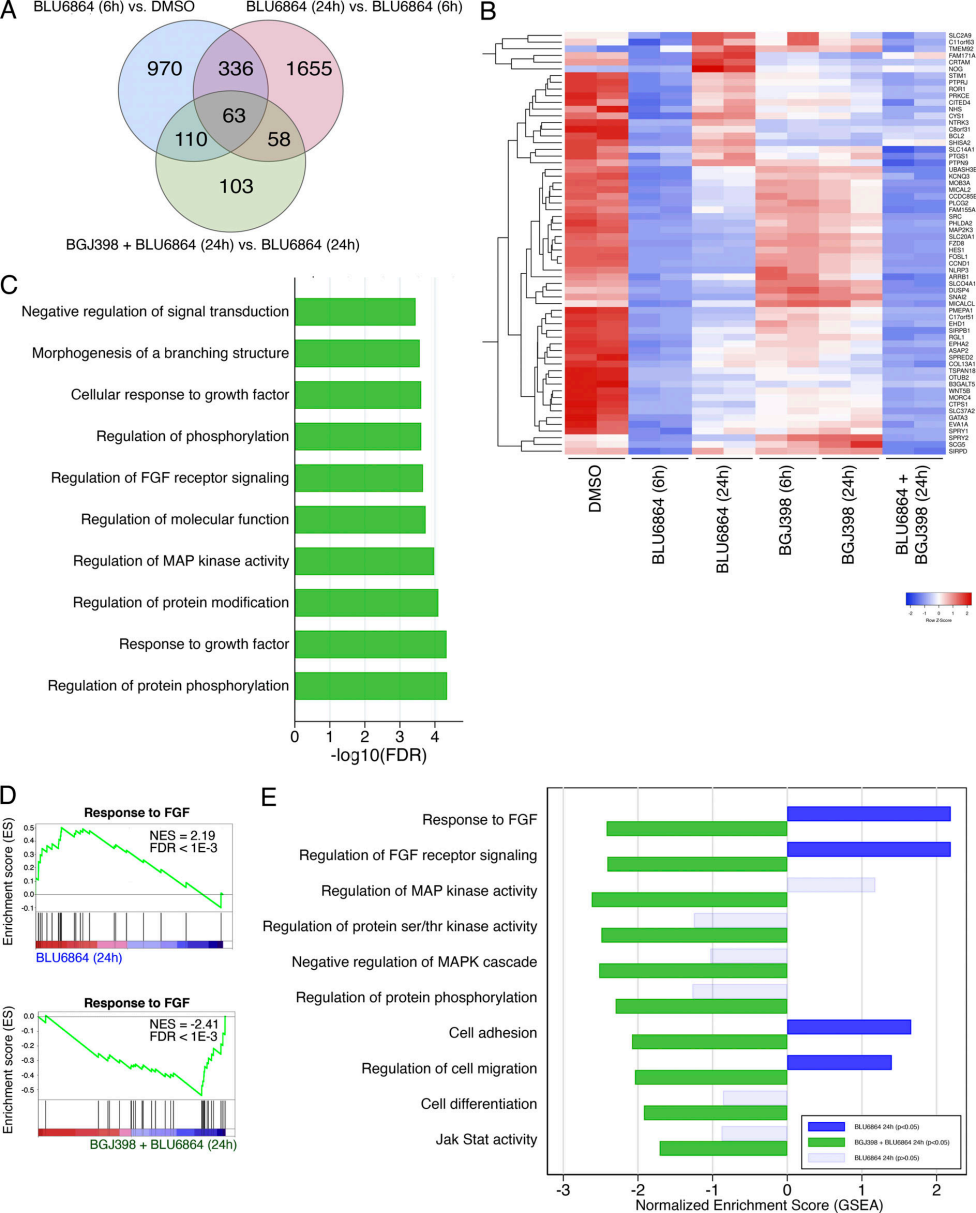
**Gene expression analysis of TPC1 cells treated with a RET inhibitor identifies adaptive resistance signature. (A)** Venn overlap of differential gene expression in TPC1 cells treated with the indicated drug combinations. **(A)** Set of 63 genes identified as correlated with adaptive resistance to BLU6864 and sensitivity to the combination of BLU6864 and BGJ398. **(B)** Heatmap of 63 gene resistance signature across treatment conditions. **(C)** Top differentially regulated pathways from 63 gene resistance signatures identified using gProfiler. **(D)** GSEA using response to FGF as a signature in BLU6864 (24 h) vs. BLU6864 (6 h; top), compared with BGJ398 + BLU6864 (24 h) vs. BLU6864 (24 h; bottom). NES, normalized enrichment score. **(E)** NESs from GSEA plotted for selected GO signatures. Differential gene expression from BLU6864 (24 h) vs. BLU6864 (6 h) is plotted in blue and compared with BGJ398 + BLU6864 (24 h) vs. BLU6864 (24 h), plotted in green. Light blue shading indicates a nonsignificant result (P > 0.05, FDR > 0.2), and dark blue shading indicates a significant result (P < 0.05, FDR < 0.2). Statistical tests were performed using the GSEA software.

We performed next-generation RNA sequencing on TPC1 cells treated with BLU6864 and BGJ398 to identify a gene expression signature of adaptive resistance to RET inhibition. We identified genes whose expression is down-regulated after exposure to BLU6864 but rebounds as resistance develops (Fig. 5 A; *n* = 399). A subset of these genes were sensitive to combined treatment with BGJ398 (*n* = 63), and we defined these as a signature of FGFR-dependent adaptive resistance to RET inhibition (Fig. 5 B). Genes induced in adaptive resistance included *SPRY1/2* and *DUSP4*, well-known negative regulators of ERK signaling (Casci et al., 1999; Guan and Butch, 1995; King et al., 1995). Using the resistance signature, we found significant enrichment in pathways associated with protein phosphorylation, MAPK activity, and FGFR signaling (Fig. 5 C). Using gene set enrichment analysis (GSEA), we performed a supervised analysis of FGFR and MAPK pathway activation in resistant (BLU6864 [24 h] vs. BLU6864 [6 h]) versus sensitive (BGJ398 + BLU6864 [24 h] vs. BLU6864 [24 h]) cells (Fig. 5 D). We found that resistant cells show marked activation of FGFR signaling that is reversed with combined treatment (Fig. 5 E). We identified additional signaling pathways that are differentially inhibited after combined treatment, including JAK/STAT signaling. These data indicate that combined treatment with BGJ398 and BLU6864 is associated with significant changes in gene expression in pathways associated with FGFR signaling, MAPK activity, and gene expression signatures linked to cell adhesion and migration.

**Figure 6.**
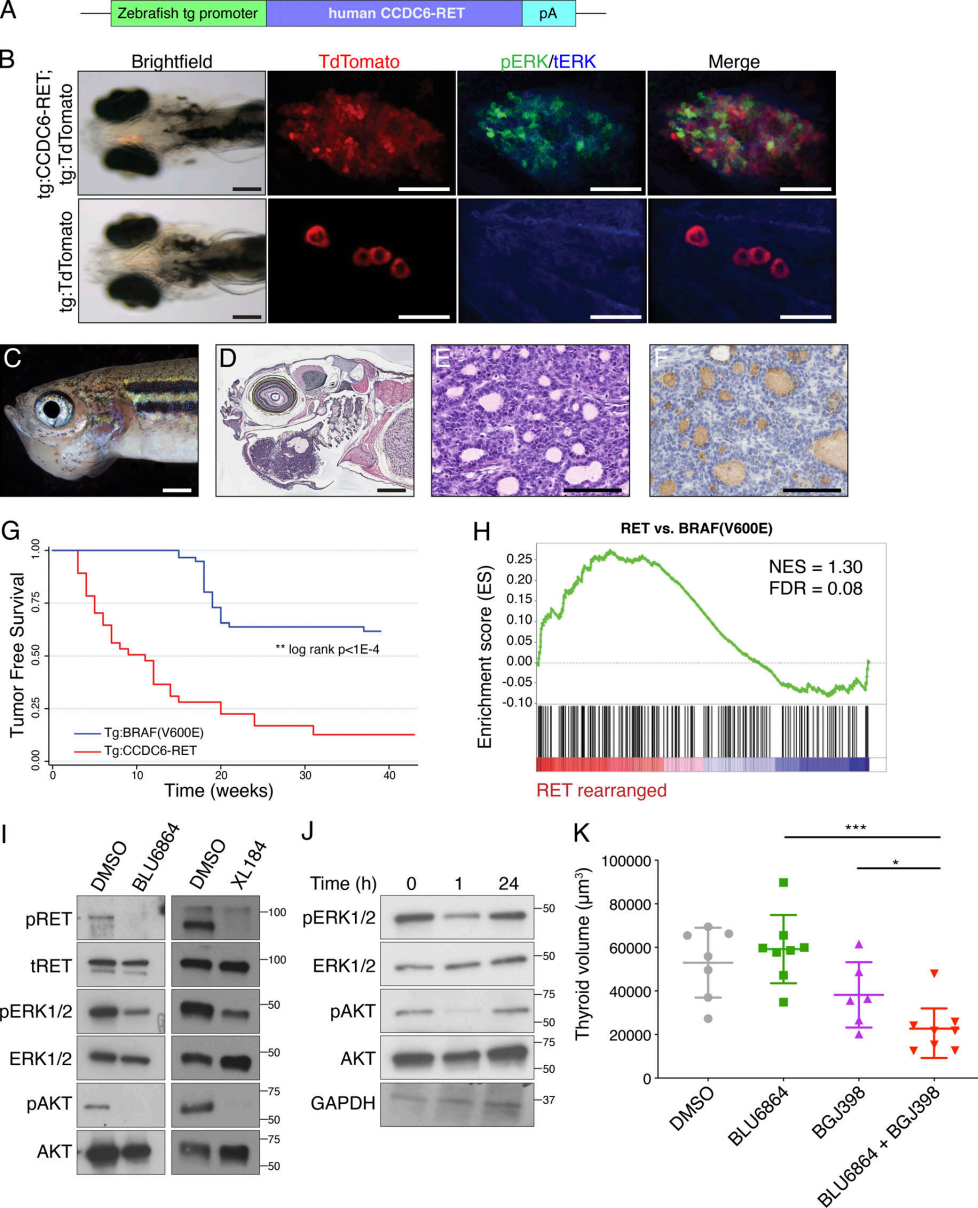
**Adaptive resistance and efficacy of combined treatment with RET and FGFR inhibitors in a transgenic model of RET-rearranged thyroid cancer. (A)** Schematic of tol2 transgene constructed to express *CCDC6-RET* fusion under control of a zebrafish thyroglobulin promoter (tg). **(B)** Fluorescence imaging of transgenic larval zebrafish at 7 dpf. Expression of *CCDC6-RET* (top) compared with wild-type thyroid (bottom). Thyroid cells are visualized in follicles and express TdTomato. ERK activation analyzed by whole-mount in situ fluorescence microscopy, pERK (green) and tERK (blue). Scale bar for bright-field images = 2 mm; scale bar for fluorescence confocal = 100 μm. Four transgenic larvae were imaged, and a representative animal is shown. **(C and D)** Juvenile zebrafish (28 dpf) with mass protruding from ventral jaw (C) and corresponding histopathologic section (D), H&E stained. **(A)** Representative tumor-bearing animal is shown from a clutch of 28 tumor-bearing animals; scale bar = 5 mm. **(E)** High-resolution photomicrograph from *RET* transgenic zebrafish tumor; scale bar = 100 μm. This image is also used in Fig. S4 H. **(F)** Immunohistochemical staining for thyroglobulin performed on a *RET* transgenic zebrafish tumor, scale bar = 100 μm. **(G)** Tumor-free survival curve comparing transgenic *RET* zebrafish (red, *n* = 110) to transgenic *BRAF(V600E);p53(M214K)*; blue, *n* = 37) zebrafish. **(A)** log-rank test was used to compare tumor-free survival; **, P < 1 × 10^−4^. **(H)** GSEA using a zebrafish *RET* gene signature (*n* = 178 genes; Table S1) and examining papillary thyroid cancers (TCGA) stratified by *RET*-rearranged status in the TCGA cohort. Statistical tests were performed using the GSEA software. **(I)** Western blot analysis of phospho-RET, phospho-ERK, and phospho-AKT in lysates harvested from tumors exposed to BLU6864 or XL184. **(J)** Western blot analysis of ERK activation in lysates prepared from zebrafish tumors after treatment with RET inhibitors over time. A representative Western blot is shown from three independent experiments. **(K)** Comparison of thyroid volume from *RET* transgenic animals treated with BLU6864, BGJ398, or the combination. Larval zebrafish were treated from 3 to 12 dpf, and thyroid volume was measured using confocal microscopy. The mean and SD are displayed. Each transgenic animal is represented by a symbol. Statistical significance was assessed by Student’s *t* test; *, P < 0.01; ***, P < 0.001.

**Figure S4.**
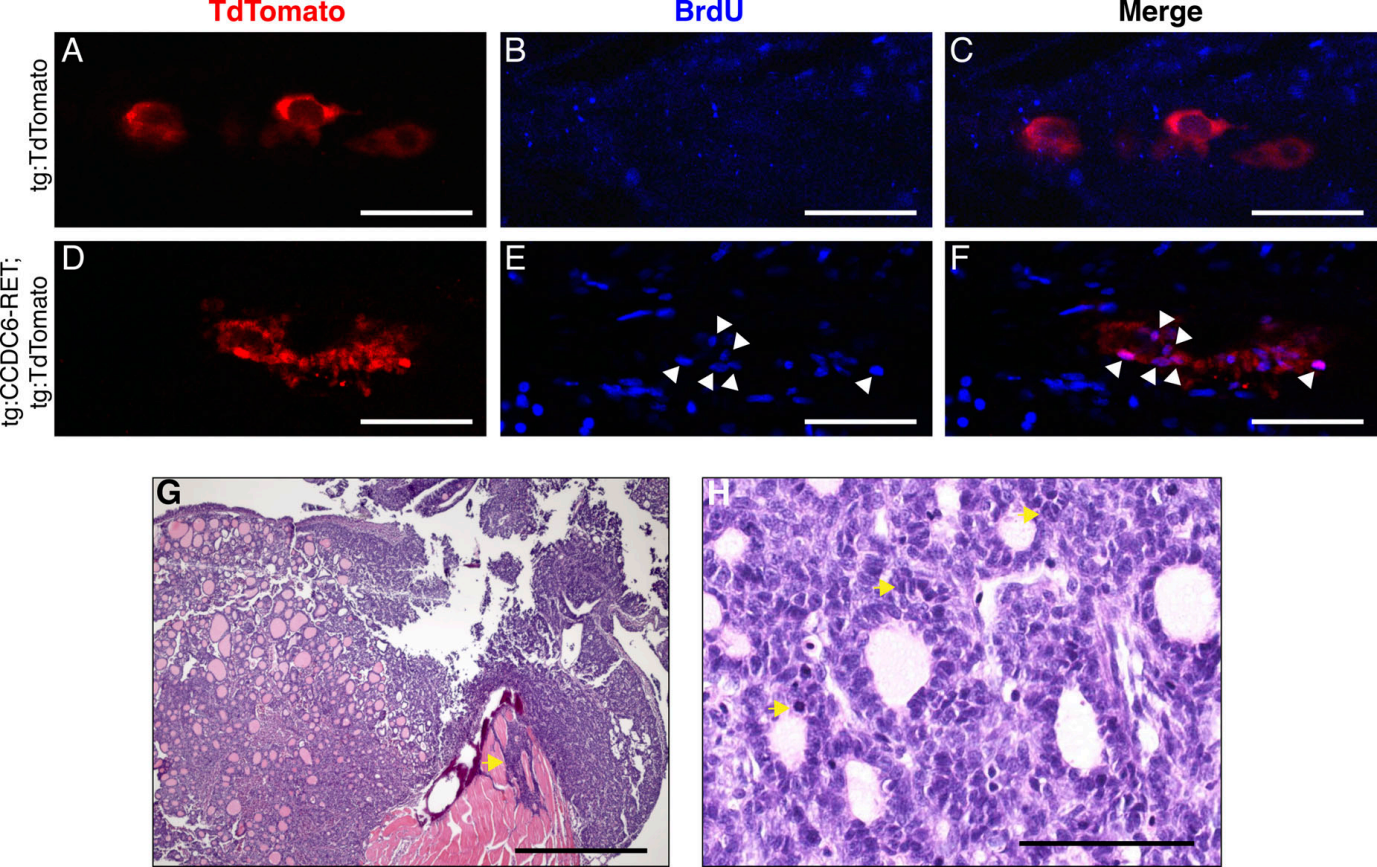
**Characterization of larval thyrocyte proliferation in transgenic RET thyroid, and histologic characterization of thyroid tumors arising in transgenic RET adult zebrafish. (A–F)** Transgenic zebrafish tg:*TdTomato* (A–C) were compared to tg:*CCDC6-RET*; *tgTdTomato* (D–F) by confocal microscopy at 5 dpf. Larval animals were stained with BrDU to identify cells in S phase (B and E). Merged images (C and F) allow identification of TdTomato + thyrocytes that costained with BrdU, as noted by arrowheads. Representative images are shown. For A**–**F, scale bar = 50 μm. **(G)** H&E staining performed on tg:*CCDC6-RET* transgenic zebrafish with tumors identifies invasion of skeletal muscle (noted by arrow); scale bar = 2 mm. **(H)** High-powered photomicrograph of *CCDC6-RET* transgenic tumor identifies mitotic cells in thyroid tumor (noted by arrows); scale bar = 100 μm. This image is also used in Fig. 4 E.

We constructed a transgenic model of *RET*-rearranged thyroid cancer using genetic approaches in zebrafish. Expression of *CCDC6-RET* under the control of a thyroid-specific promoter (Fig. 6 A) led to enhanced proliferation in larval zebrafish and increased activation of ERK signaling in transgenic thyroid cells compared with wild-type thyroid cells (Fig. 6 B). We observed an increased rate of proliferation in transgenic thyroid cells after staining with BrdU (Fig. S4, A–F). Larval zebrafish grew into juvenile fish with visible masses protruding from the ventral jaw (Fig. 6, C and D) and histologic features of thyroid carcinoma including nuclear grooves and mitotic figures (Fig. 6, E and F). *RET* transgenic tumors displayed invasion of skeletal muscle (Fig. S4, G and H). Transgenic *RET* zebrafish developed tumors at a significantly increased rate as compared with a BRAF(V600E) model of thyroid cancer (Fig. 6 G; Anelli et al., 2017). We performed next-generation RNA sequencing of *RET* zebrafish tumors to determine effects on gene expression. By comparing gene expression in transgenic *RET* and *BRAF(V600E)* thyroid tumors, we identified a zebrafish *RET* gene signature (*n* = 178 genes; Table S1). We sought to determine whether this signature was conserved in human thyroid cancers harboring *RET* rearrangements. Using the TCGA thyroid cancer cohort, we found that the zebrafish *RET* gene signature is enriched in human thyroid cancers that harbor *RET* rearrangements (Fig. 6 H), consistent with a conservation of gene expression in the zebrafish model.

We next examined the sensitivity of transgenic *RET* zebrafish tumors to RET inhibitors BLU6864 and XL184. Ex vivo treatment of zebrafish *RET* tumors with BLU6864 or XL184 was associated with inhibition of pRET, pERK, and pAKT, consistent with an on-target effect. A time-course analysis of ERK signaling after treatment with BLU6864 revealed a rebound in pERK and pAKT at 24 h, consistent with development of adaptive resistance. We examined the consequence of drug treatment on the growth of thyroid masses in transgenic larval zebrafish using confocal microscopy. Short-term treatment with either BLU6864 or BGJ398 alone was associated with modest effects on thyroid volume. Combined treatment with BLU6864 and BGJ398 was associated with a significant decrease in thyroid volume compared with either drug alone (Fig. 6 I), consistent with a synergistic effect on cell growth and viability. Using zebrafish we built a patient-specific avatar of *RET*-rearranged thyroid cancer and demonstrated conservation of gene expression, sensitivity to RET inhibition, and synergy between RET and FGFR inhibitors in vivo.

## Discussion

Inhibition of ERK signaling poses unique challenges, as disruption of homeostatic mechanisms that negatively regulate MAPK activity lead to paradoxical rebound activation (Sun and Bernards, 2014). In *CCDC6-RET*–rearranged thyroid cancer cells, we found activation of ERK signaling after RET inhibition. In our studies, ERK signaling and cellular growth were insensitive to BGJ398 inhibition in TPC1 cells before RET inhibition. Our data are consistent with other studies demonstrating that RET signaling is the principal input into ERK activation in TPC1 cells (Mologni et al., 2006). Cross talk between ERK and FGFR signaling has been shown to act through several mechanisms, including inhibitory phosphorylation of threonine residues on FRS2 (Lax et al., 2002) and inhibitory phosphorylation of FGFR1 (Ser777). In cells in which full-length RET is expressed as a transmembrane protein, FRS2 has been shown to bind to pY1062 on RET (Melillo et al., 2001), although it is unknown whether this interaction occurs in cytoplasmic RET fusion proteins. We observed an FGFR-dependent increase in FRS2 phosphorylation after RET inhibition, suggesting that CCDC6-RET–expressing cells are poised to rapidly activate FGFR signaling upon RET inhibition. We also identified a transcriptional signature of adaptive resistance characterized by altered expression of *SPRY/DUSP* genes that is reversed with FGFR inhibition. Our data suggest that inhibition of RET in CCDC6-RET fusion–positive thyroid cells leads to rebound activation of ERK via an FGFR-dependent mechanism.

RET is activated as an oncogene through diverse mechanisms, including mutations affecting extracellular cysteine residues that result in ligand-independent receptor dimerization, activating mutations in the kinase domain, and chromosomal rearrangements that lead to aberrant expression of a chimeric fusion protein (Santoro et al., 2020). Point mutations in the RET kinase domain have been demonstrated to activate aberrant signaling pathways (Songyang et al., 1995), underscoring the need to examine RET mutants in genetic and cellular contexts. RET fusions have been shown to exhibit differential transforming potency and to signal through different networks depending on the fusion partner (Basolo et al., 2002; Levinson and Cagan, 2016). Further studies are needed to define the precise differences in signaling and drug resistance between RET fusions in papillary thyroid cells and RET mutants in neuroendocrine C-cells. We analyzed the TCGA papillary thyroid cancer dataset and found that *RET*-rearranged cancers have higher levels of ERK activity compared with BRAF/RAS mutant cancers, which may be the basis for a sensitivity to adaptive resistance after RET inhibition. Further studies will be required to understand the sensitivity of alternative RET fusion proteins in thyroid cancer and to examine RET fusions in other cancer contexts, including lung adenocarcinoma.

The index patient in our study offered a unique opportunity to examine recurrent disease over three decades. RET-rearranged thyroid cancer has been associated with exposure to radiation, younger age of onset, a tendency to concentrate radioactive iodine, and in some patients, an indolent clinical course. Molecular analysis from two metastatic lesions identified a *TERT* promoter mutation and loss of *CDKN2A* in the index patient. *TERT* promoter mutations have been identified in ∼9% of papillary thyroid cancers and 40–73% of advanced thyroid cancers, where they are associated with a more aggressive clinical course (Cancer Genome Atlas Research, 2014; Liu et al., 2013; Landa et al., 2016). *CDKN2A* loss has been reported in anaplastic thyroid cancer, but the frequency of this genetic alteration in RET-rearranged cancer is unknown. Our data indicate that disease progression in WCM271 was associated with the acquisition of a mutation in the *TERT* promoter. Because of limited available tumor tissue from the index patient, however, we were unable to perform studies to determine whether FGFR pathway activation was responsible for RET inhibitor resistance. In transgenic mouse models of *RET*-rearranged thyroid cancer, *RET* transgene copy number has been correlated with disease severity (Knostman et al., 2007), tumor onset has ranged from weeks to months, metastasis is uncommon (<10% on RET/PTC3), and metastasis is modestly enhanced on a *p53* mutant background. Expression of human *CCDC6-RET* in zebrafish was associated with robust thyroid proliferation, activation of ERK signaling, development of thyroid carcinoma within weeks, and adaptive resistance after exposure to a selective RET inhibitor. Interestingly, inhibition of RET in zebrafish tumor tissue was associated with a rebound of AKT pathway activation, in contrast to TPC1 cells, in which the AKT pathway remains suppressed after treatment with a selective RET inhibitor. Transgenic *RET* zebrafish larvae treated with a combination of RET and FGFR inhibitors had a significant decrease in tumor volume compared with either drug alone.

Inhibition of SHP2 has emerged as a promising approach to treat RTK-driven cancers (Chen et al., 2016), and to prevent adaptive resistance in RAS mutant cancers (Fedele et al., 2018). In KRAS(G12C)-addicted tumor models, resistance mechanisms may involve upregulation of RTKs, alternative signaling through PI3K/AKT, and SHP2 dependence, depending on cellular context (Hallin et al., 2020; Amodio et al., 2020). In *RET*-rearranged TPC1 cells, SHP2 phosphorylation increased after treatment with a RET inhibitor and remained elevated after treatment with an FGFR inhibitor. We found that ERK activity is sensitive to SHP2 inhibition, but SHP2 inhibition is not sufficient to bypass adaptive resistance arising from RET inhibition. Our data suggest that SHP2 may play a unique role in modulating ERK pathway activation in *CCDC6-RET*–rearranged thyroid cancer cells, and that pSHP2 and pSTAT3 may be biomarkers of adaptive resistance to RET inhibition.

Our data add to a growing body of literature describing a rebound in ERK signaling after treatment with kinase inhibitors. Resistance mechanisms in RET-dependent cancers may differ depending on cell type, *RET* gene mutation, RET expression level, and *TERT* promoter mutation status. Recent studies indicate that amplification of *MET* is a druggable genetic mechanism of resistance in RET fusion–positive lung cancer (Rosen et al., 2021). The identification of FGFR activation as a mechanism of resistance to RET inhibitors provides an opportunity to anticipate resistance to selective RET inhibitors and suggests that combination therapy with FGFR inhibitors may lead to more significant and durable antitumor responses.

## Materials and methods

### FISH

5-μm-thick formalin-fixed, paraffin-embedded tissue sections were cut for FISH analysis. *RET* break-apart was validated using dual-color FISH probes (RP11-89J23 BAC clone labeled red; RP11-379D20 labeled green). *RET* break-apart was determined as one individual green signal and one individual red signal, per nucleus. Before use, all clones were validated on metaphase spreads. A minimum of 100 nuclei were observed per slide using a fluorescence microscope (Olympus BX51; Olympus Optical). Cytovision and Fiji software were used for image analysis.

### RNA sequencing and analysis

Total RNA was extracted from fresh frozen tissue (WCM271) using a Maxwell 16 LEV simplyRNA purification kit (Promega) in conjunction with a Maxwell 16 MDx instrument (Promega). RNA integrity was verified using a Bioanalyzer 2100 (Agilent Technologies). Libraries were prepared using TruSeq stranded library preparation kit (Illumina) and sequenced on a paired-end read flow cell for 100 cycles on an Illumina HiSeq2000 (Illumina). FASTQ files were mapped to human genome build hg38 using STAR (v2.4.01f1), and gene expression values (FPKM) were estimated with Cufflinks (v2.0.2). TCGA expression data was downloaded (https://portal.gdc.cancer.gov/) and batch normalized (ComBat) to permit comparison with WCM271. Gene expression values for 16 thyroid function genes (DIO1, DIO2, DUOX1, DUOX2, FOXE1, GLIS3, NKX2-1, PAX8, SLC26A4, SLC5A5, SLC5A8, TG, THRA, THRB, TPO, and TSHR) were used to calculate the thyroid differentiation score as described (Cancer Genome Atlas Research, 2014). Gene fusions from TCGA were detected using STAR-fusion (v0.5.1) and validated with published data (Gao et al., 2018).

### Analysis of cfDNA

cfDNA was isolated from plasma using the QIAamp Circulating Nucleic Acid Kit (Qiagen), with cfDNA concentration assessed using the Qubit 3.0 Fluorometer (Thermo Fisher Scientific). cfDNA was prepared for next-generation sequencing analyses using the PGDx elio plasma resolve assay (Personal Genome Diagnostics). 40 ng of cfDNA was prepared through genomic library preparation and in-solution hybrid capture. Next-generation sequencing was performed using the NextSeq 550 (Illumina) with NextSeq 500/550 High Output v2 (150 cycles) sequencing. Data analyses were preformed using the PGDx elio plasma resolve bioinformatics pipeline, previously developed using an independent training cohort for the establishment of variant filtering thresholds. Briefly, base calls were demultiplexed using Picard (v2.8.14), and sequencing reads were aligned to the hg 19 reference genome using BWA (v0.7.15) and Bowtie2 (v2.3.1). Genetic alterations were identified using VariantDx, and a custom analysis pipeline was used to identify gene rearrangements and estimate allele frequencies.

### Archival tissue genotyping

DNA was isolated from archival tumor formalin-fixed, paraffin-embedded samples and sequenced using the hybridization-capture based TruSight Oncology 500 panel (TSO500; Illumina). Sequencing libraries were prepared from 100 ng of extracted tumor DNA according to the Illumina TruSight Oncology 500 Reference Guide (Illumina). Quantification and quality control of the sequencing library were performed using the Qubit DNA HS Assay Kit and Qubit 2.0 fluorometer (Invitrogen). Libraries were pooled and sequenced on the NextSeq 550DX System (Illumina) using the NextSeq High Output v2.5 reagent kit (Illumina). The sequencing data were analyzed using the TruSight Pipeline v2.2 (Illumina) and reviewed using the Clinical Genomics Workspace (PierianDx). Manual review of the BAM files was also performed for detailed analysis of the TERT promoter region.

### High-throughput drug screening

In collaboration with SEngine, we performed drug screens on TPC1 cells. TPC1 cells were expanded in vitro and assessed for viability immediately before high-throughput screening profiling. 500 cells were seeded per well into 384-well assay plates containing 50 µl growth medium and allowed to attach overnight. A 209 small-molecule drug library was acoustically administered (Labcyte Echo) in a randomized pattern to individual wells as single agents using contactless liquid transfers to create a 3-log, 6-dose drug curve, as previously described (Narasimhan et al., 2020). Drug concentrations ranged from 1 nM to 10 μM. Dose ranges were designed to capture previously reported *C*_max_ values and the asymptotic response range. Cellular viability was assessed 6 d after drug addition using CellTiter-Glo (Promega). Dose–response curves were generated for each drug using a five-parameter logistic fit model, and area under the curve was calculated. The dose response of TPC1 cells treated with kinase inhibitors was compared with the Cure First/SEngine Precision Medicine database of 53 primary tumor samples representing multiple tumor types, generating Z-score values, as previously described (Pauli et al., 2017).

### Western blots

TPC1 cells (catalog SCC147; Sigma-Aldrich) were grown in DMEM supplemented with 10% FBS and plated at 50% confluency 1 d before drug treatment. CUTC48 cells (University of Colorado’s Cell Culture Services Core) were cultured in Copland medium supplemented with 10% FBS and plated at 50% confluency 1 d before drug treatment. Protein extracts were prepared using radioimmunoprecipitation assay buffer supplemented with Halt protease/phosphatase inhibitors (PI78442; VWR). Antibodies used for Western blot analysis were anti-pERK (T202/Y204; Cell Signaling Technology, #9101), anti-ERK (ERK1/2; Cell Signaling Technology, #9102), anti-pRET (Y905; Cell Signaling Technology, #3221), anti-RET (C31B4; Cell Signaling Technology, #3223), anti-pAKT (S473; Cell Signaling Technology, #D9E), anti-AKT (C67E7; Cell Signaling Technology, #4691), anti-pSTAT3 (Cell Signaling Technology, #9131), anti-STAT3 (Cell Signaling Technology, #4904), anti-pSHP2 (Y542; Cell Signaling Technology, #3751), anti-SHP2 (D50F2; Cell Signaling Technology, #3397), anti-pFRS2a (Y196; Cell Signaling Technology, #3864), anti-FRS2 (R&D Systems, #MAB4069), anti-GAPDH (Cell Signaling Technology, #D16H11), and anti-FGFR1 (Cell Signaling Technology, #9740T). BLU6864 was provided by Blueprint Medicines.

### Phosphotyrosine proteomics and mass spectrometry

TPC1 cells were treated with 100 nM BLU6864 or vehicle for 24 h. Cells were washed three times with ice-cold PBS, scraped, pelleted by centrifugation, and snap frozen in liquid nitrogen. Thawed cell pellets were lysed by sonicating in 9 M urea, 50 mM ammonium bicarbonate, 1 mM NaVO_4_, 1 mM β-glycerophosphate, and 2.5 mM Na_4_P_2_O_7_. Insoluble material was removed via centrifugation. Proteins were reduced and alkylated with dithiothreitol and iodoacetamide and digested overnight with LysC after diluting with 2 M urea and for 6 h at 37°C with trypsin. Peptide were desalted by C18 solid-phase extraction, and phosphotyrosine peptides were enriched using the P-Tyr-1000 kit (Cell Signaling Technology, #8803). Samples were analyzed on a Thermo Orbitrap Fusion mass spectrometer. MS1 scans were performed in the orbitrap at 120 K. The top *n*-most intense peaks in a 2-s cycle time were selected for isolation, collision-induced dissociation fragmentation, and MS/MS analysis in the linear ion trap. Spectra were searched against a composite database of all canonical human protein sequences and their reversed complement using SEQUEST v28 (rev. 13; Eng et al., 1994). Search parameters allowed three missed cleavages, a 20-ppm mass error, a static modification of 57.02146 daltons (carboxyamidomethylation) on Cys, and dynamic modifications of 15.99491 daltons (oxidation) on Met and 79.96633 on Ser, Thr, and Tyr. Spectral matches were filtered to 5% false discovery rate (FDR) using the target-decoy strategy (Elias and Gygi, 2007) and linear discriminant analysis (Huttlin et al., 2010). The data were further filtered to require a peak signal-to-noise value ≥5. The final peptide FDR was <0.5%. Label-free peptide intensities were derived from the integrated area under the curve of precursor MS1 peaks. Quantitative comparisons were made to identical phosphopeptide species, defined by primary sequence, charge, *m*/*z*, and matching numbers of all modifications. Phosphorylation site assignment was performed using the Ascore algorithm (Beausoleil et al., 2006).

### Cellular proliferation assays

5,000 TPC1 cells were treated in 96-well format in DMEM supplemented with 1% FBS. Individual drugs were diluted in medium with 0.1% DMSO. Drug-containing medium was replaced at 48 h. Proliferation was measured at 96 h using CellTiter-Glo (Promega), and Prism (GraphPad) was used to fit the data, generate dose–response curves, and calculate EC_50_ values. For colony formation assays, 500 TPC1 cells were seeded in 6-well plates in DMEM supplemented with 10% FBS. Individual drugs were diluted in medium with 0.1% DMSO. Medium containing drugs was refreshed every 48 h. Crystal violet staining was performed after 7 d. Crystal violet–stained plates were photographed, and ImageJ software was used to quantify colony intensity percentage for each condition.

### Lentiviral infection and FGFR1 gene targeting

Lentiviral infection and CRISPR/Cas9 genome editing were used to generate TPC1 clones with targeted disruption of FGFR1 coding sequence. A Cas9 encoding lentivirus was generated by transfection of HEK293T cells using plasmids encoding pLentiV-Cas9-P2A-Puro, psPAX2, and pCMV-VSV-G. TPC1 cells expressing Cas9 (TPC1-Cas9) were isolated by puromycin selection and expanded. TPC1-Cas9 cells were subsequently infected with lentivirus (VectorBuilder gRNA#7940) encoding a sgRNA targeting FGFR1 (5′-GCC​ACT​TTG​GTC​ACA​CGG​TTG​GG-3′; NCBI Gene ID: 2260). 200 µl of 10× concentrated virus was transduced into TPC1-Cas9 cells to obtain cells containing edits in the FGFR1 gene. Individual clones were isolated and expanded for biochemical studies and colony formation. Confirmation of FGFR1 targeting was performed by Sanger sequencing of individual clones isolated after TOPO TA cloning (Invitrogen; PCR products flanking the sgRNA site: forward, 5′-CGG​GAC​AGA​CTG​GTC​TTA​GG-3′; reverse, 5′- GCT​TCC​CGA​TCA​TCT​TCA​TC-3′).

### Differential gene expression and pathway analysis

TPC1 cells were treated with vehicle (DMSO), 100 nM BLU6864, 100 nM BGJ398, or the combination. RNA was isolated from treated cells using RNeasy Mini Kits (Qiagen), and library preparation was performed as described above. Libraries were sequenced on a paired-end flow cell for 100 cycles using an Illumina HiSeq 2500. Alignment was performed using Star v2.7.7 (Dobin et al., 2013), and differential gene expression was performed with DeSeq2 (Love et al., 2014). Differentially expressed genes were identified with log2 fold-change < −0.5 and P < 0.01 in each comparison. g:Profiler (Raudvere et al., 2019) was used to identify differentially regulated pathways associated with adaptive resistance signature genes. GSEA (Subramanian et al., 2005) was performed using Gene Ontology (GO) signatures, and the top 3,000 differentially expressed genes were sorted by log2 fold-change using a rank ordered list.

### Zebrafish transgenesis, tumor modeling, and gene expression

Human CCDC6-RET was PCR amplified and sequence verified from pEGFP-C1-CCDC6-RET (Medical Research Council, University of Dundee) and cloned using Gateway recombination (Life Technologies) as a middle entry clone. A 514-bp fragment of the zebrafish thyroglobulin promoter (tg) was used as a 5′ clone, and a p3E-polyA was used as a 3′ clone, as previously described (Anelli et al., 2017). 25 pg of sequence-verified pTol2-tg:CCDC6-RET-pA was injected into tg:TdTomato embryos. Embryos were raised to adulthood and outcrossed. F1 transgenic animals were screened by PCR for the presence of a CCDC6-RET transgene to identify founders. BrdU incorporation was performed as previously described (Anelli et al., 2017). A zebrafish tg:BRAF(V600E) thyroid cancer model was previously described (Anelli et al., 2017). Cohorts of tg:CCDC6-RET or tg:BRAF(V600E) transgenic zebrafish were followed weekly to determine the rate of tumor formation. Transgenic animals with visible masses were isolated, and tumors were analyzed by histopathology to confirm the presence of thyroid cancer, as described (Anelli et al., 2017). RNA was isolated from transgenic BRAF(V600E) or CCDC6-RET tumors and subjected to library preparation and next-generation sequencing. Libraries were sequenced on a paired-end flow cell for 100 cycles using an Illumina HiSeq2500. Alignment was performed using Star v2.7.7 (Dobin et al., 2013), and differential gene expression was performed with DeSeq2 (Love et al., 2014). A zebrafish RET gene signature was derived by comparing gene expression in transgenic RET zebrafish thyroid tumors (tg:CCDC6-RET) to transgenic BRAF(V600E) zebrafish tumors (tg:BRAF(V600E)). 178 human orthologs with log2 fold-change >4 and P < 1 × 10^−3^ (DeSeq2) were identified (Table S1) and used in downstream GSEA. GSEA was performed using TCGA thyroid samples stratified by RET rearrangement status.

### Inhibitor treatment and thyroid volume measurement in larval zebrafish

*Tg:EGFP-CCDC6-RET;Tg:TdTomato* larvae were incubated at 3 d postfertilization (dpf) in 2 ml E3 fish water containing 0.1% DMSO in a 6-well plate in the presence of 5 μM BGJ398 (Selleckchem), 1 μM BLU6864 (Blueprint Medicines), or a combination of the two inhibitors. These doses were tolerated without gross morphologic or developmental defects. Fresh medium containing drug was replaced at 7 and 10 dpf. At 12 dpf, larvae were anesthetized in 0.04% Tricaine-S (Pentair) and transferred to 4% methylcellulose/E3 in a 35-mm dish with a coverslip bottom (MatTek). TdTomato-positive thyroid cells were imaged using a Zeiss LSM800 confocal with a Plan Apochromat 20×/0.75 objective lens, and 3D tumor volume was determined using Imaris (BitPlane) software.

### Ethics

Animal studies were performed in strict accordance with the recommendations in the Guide for the Care and Use of Laboratory Animals of the National Institutes of Health. Zebrafish were handled according to an approved Institutional Animal Care and Use Committee protocol (#2011-0026) of Weill Cornell Medical College. WCM271 provided informed consent as a study subject in protocols 1305013903, 1007011157, which were reviewed and approved by Weill Cornell Medical College’s Institutional Review Board.

### Online supplemental material

**Figure S5.**
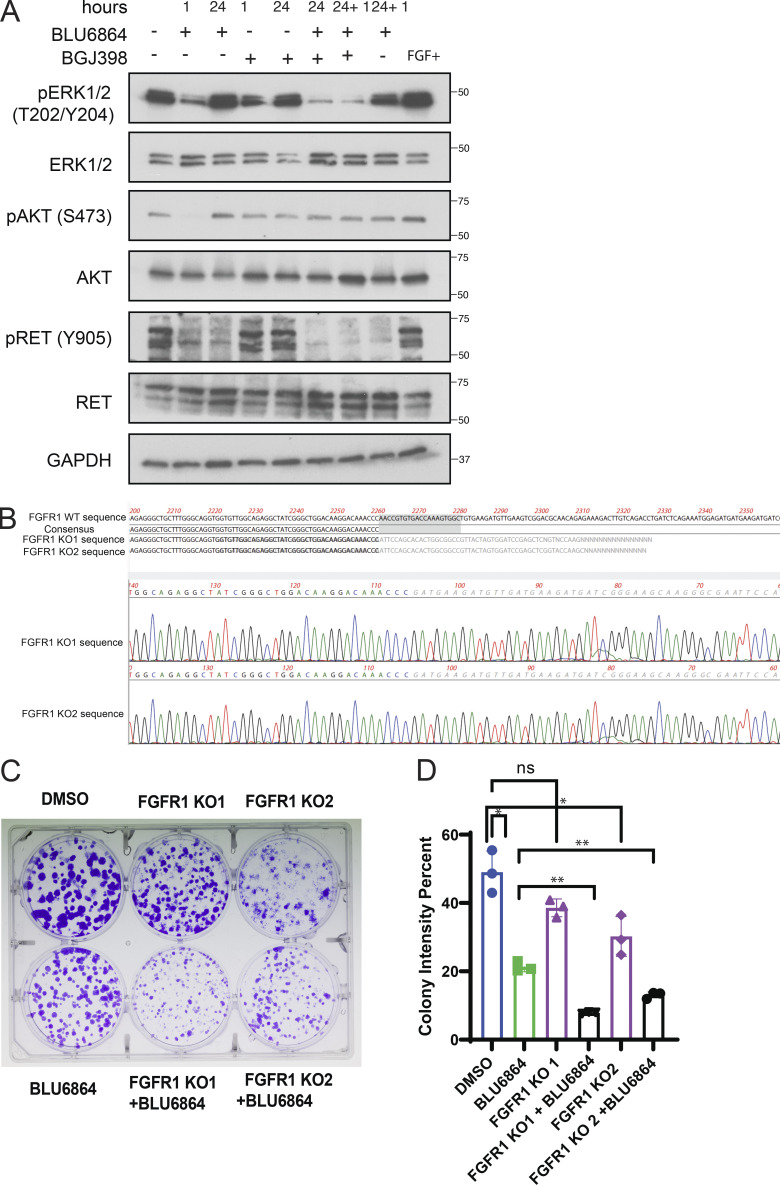
**Adaptive resistance in RET fusion thyroid cancer cell line CUTC48; genetic inactivation of FGFR1 abrogates adaptive resistance to RET inhibitors. (A)** Western blot analysis of ERK activation and downstream signaling effectors in CUTC48 cells treated with BLU6864 alone or in combination with BGJ398 over the indicated time course. A 24 + 1 timepoint indicates that the BGJ398 was added for 1 h after an initial incubation in BLU6864 for 24 h. CUTC48 cells were obtained from the University of Colorado’s Cell Culture Services Core. **(B)** Sanger sequencing of individual clones isolated from lentiviral-mediated CRISPR-Cas9 gene-edited TPC1 cells shows genomic deletion of FGFR1 gene in two different FGFR1 knockout clones. **(C)** Long-term proliferation assay of FGFR1 KO TPC1 cells treated with XL184 or BLU6864 alone or in combination with BGJ398. Cells were treated for 12 d and stained with crystal violet. **(D)** Quantification of long-term viability of FGFR1 KO TPC1 cells treated with the indicated compounds was performed by calculating colony intensity; mean and SD are displayed for technical replicates from one of three biological replicates. *, P < 0.05; **, P < 0.01, Student’s *t* test.

Fig. S1 presents additional data regarding genetic alterations identified in WCM271, and immunohistochemistry from primary tumor cells grown in culture. Fig. S2 shows ERK score from papillary thyroid cancers in the TCGA cohort stratified by RET rearrangement, BRAF mutant, or HRAS/NRAS mutant. Fig. S3 presents biochemical data on ERK pathway activation in MZCRC1 and TT cells after treatment with RET and FGFR inhibitors. Fig. S4 shows characterization of thyroid cellular proliferation in a zebrafish RET transgenic model and characterization of thyroid tumors arising in adult RET transgenic zebrafish. Fig. S5 presents biochemical response to RET inhibitors in CUTC48 cells, DNA sequencing of FGFR1 in TPC1 clones after gene editing, and proliferation of TPC1 cells harboring an FGFR1 loss of function mutation after treatment with RET and FGFR inhibitors. Table S1 lists genes identified as a RET signature.

## Supplementary Material

Table S1 lists genes identified as an RET signature.

## Data Availability

The raw and processed data from the RNA-seq experiments has been deposited in the Gene Expression Omnibus archive and can be found using the following accession numbers: GSE199022 is a superseries encompassing experiments performed in human TPC1 cells, and GSE199023 represents data from transgenic zebrafish model experiments.
